# Detecting disabilities in everyday life: evidence from a geriatric assessment

**DOI:** 10.1186/s12877-022-03368-x

**Published:** 2022-08-31

**Authors:** Cornelius Dzien, Petra Unterberger, Paul Hofmarcher, Hannes Winner, Monika Lechleitner

**Affiliations:** 1 Landeskrankenhaus Hochzirl – Natters, In der Stille 20, Natters, 6161 Austria; 2 University of Salzburg, Mönchsberg 2a, Salzburg, 5020 Austria

**Keywords:** Geriatric assessment, Functional limitations, Activities of daily living (ADL), Variable selection, Least absolute shrinkage and selection operator (LASSO), Receiver operating characteristic (ROC) analysis

## Abstract

**Background:**

The activities of daily living (ADL) score is a widely used index to establish the degree of independence from any help in everyday life situations. Measuring ADL accurately is time-consuming and costly. This paper presents a framework to approximate ADL via variables usually collected in comprehensive geriatric assessments. We show that the selected variables serve as good indicators in explaining the physical disabilities of older patients.

**Methods:**

Our sample included information from a geriatric assessment of 326 patients aged between 64 and 99 years in a hospital in Tyrol, Austria. In addition to ADL, 23 variables reflecting the physical and mental status of these patients were recorded during the assessment. We performed least absolute shrinkage and selection operator (LASSO) to determine which of these variables had the highest impact on explaining ADL. Then, we used receiver operating characteristic (ROC) analysis and logistic regression techniques to validate our model performance. Finally, we calculated cut-off points for each of the selected variables to show the values at which ADL fall below a certain threshold.

**Results:**

Mobility, urinary incontinence, nutritional status and cognitive function were most closely related to ADL and, therefore, to geriatric patients’ functional limitations. Jointly, the selected variables were able to detect neediness with high accuracy (area under the ROC curve (AUC) = 0.89 and 0.91, respectively). If a patient had a limitation in one of these variables, the probability of everyday life disability increased with a statistically significant factor between 2.4 (nutritional status, 95%-CI 1.5–3.9) and 15.1 (urinary incontinence, 95%-CI 3.6–63.4).

**Conclusions:**

Our study highlights the most important impairments of everyday life to facilitate more efficient use of clinical resources, which in turn allows for more targeted treatment of geriatric patients. At the patient level, our approach enables early detection of functional limitations and timely indications of a possible need for assistance in everyday life.

**Supplementary Information:**

The online version contains supplementary material available at (10.1186/s12877-022-03368-x).

## Background

Many countries around the world are faced with rapidly aging societies. This demographic shift not only poses capacity problems for health care providers but also changes the quality-related requirements for health services. Early detection of diseases and impairments of everyday life is therefore an indispensable task to maintain the sustainability of the healthcare system.

This study addresses the mental and physical impairments of older people who underwent geriatric screening to assess their health status and potential limitations to participation in daily life. Such assessments typically rely on formal tools such as questionnaires and tests to evaluate the physical and mental dimensions of older adults’ health [1–3]. A key issue is the measurement of functional impairments, i.e., a person’s inability to perform activities needed in everyday life. In clinical practice, the activities of daily living (ADL) score is used to measure such limitations. ADL is part of the geriatric assessment and represents a compound index of different everyday tasks, such as the ability to toilet, bathe or dress. The collection of these components requires ongoing inpatient observation and queries to the referring physician as well as large amounts of bureaucratic work. Providing an accurate and comprehensive measure of ADL is therefore a time-consuming and costly task. Hence, using more easily available information that is also collected in geriatric assessments to approximate ADL may facilitate a more efficient use of clinical resources, which in turn may improve the treatment of geriatric patients.

Previous research has adopted three major approaches to approximate ADL [4]. A first strand of literature used sensor data from wearable devices or smart home environments to obtain information on functional limitations [5, 6]. Although these studies provided accurate results, they were faced with specific problems, especially in the older and population. For example, not everyone in this age cohort is able to operate electronic devices properly. A second line of research relied on large-scale data and machine learning algorithms to predict ADL. For example, Wojtusiak et al. used patient records of approximately 200,000 patients in the US and identified 578 patient characteristics to explain ADL [4]. In addition to the fact that comparable datasets are not easily available, such a large number of explanatory variables is hardly suitable for daily clinical use, particularly in the outpatient sector. Therefore, a third approach referred to clinical data (e.g., from patient admissions), which are more easily available and also have a high degree of accuracy. For instance, Guralnik et al. and Prasitsiriphon and Weber utilized large-scale clinical studies from the US and Thailand and identified mainly physical performance measures influencing ADL [7, 8]. In a similar vein, Jonkman et al. conducted a cohort study on patients aged between 65 and 75 years in four countries (Germany, UK, Italy and the Netherlands) and identified ten out of 22 potential covariates to predict ADL.

However, the participants did not suffer from an ADL disability at baseline and only 22.3% developed one in the following three years. One possible reason for this could be the fact that only participants younger than 75 years were included in the study. The authors concluded that additional research with adults above 75 years was required [9].

This paper is related to the third strand of literature and extends the existing research in two ways. First, using clinical data from a comprehensive assessment of 326 geriatric patients, we focused on the age group between 64 and 99 years. Hence, we were particularly able to detect ADL for older-aged patients. Second, we propose a framework that allows us to not only identify the most important explanatory variables of ADL but also to estimate the cut-off values at which ADL falls below a certain threshold. Monitoring the selected explanatory variables together with their derived cut-off values could be interesting from a clinical perspective as it supports more efficient use of medical resources, which in turn allows for more targeted treatment of geriatric patients. In addition, it facilitates early detection of functional limitations and the potential need for daily assistance, which might be particularly useful in outpatient care.

## Methods

To explain ADL of older patients, we conducted a cross-sectional study in the hospital of Hochzirl, Tyrol (Austria). This hospital has its own geriatric ward and is accordingly specialised in the care of geriatric patients. Patients are referred either by an outpatient centre (general practitioner), by clinics (e.g. infectious diseases, cardiology or traumatology) or by emergency departments of related hospitals, as the hospital of Hochzirl does not have its own emergency department. A medical report was prepared for each patient at the time of registration. This report was based on the medical history provided by the referring facility, a medical admission interview and a physical examination. In addition, older adults underwent a comprehensive geriatric assessment, including a variety of screening tests and the elicitation of anthropometric data. Overall, information was gathered on general health status, mobility, cognitive ability, nutritional status, urinary incontinence, sensory functions and psychological situation. Furthermore, ADL data were collected for each patient.

### Data

Our sample included 326 patients aged between 64 and 99 years who underwent geriatric assessment between July 2019 and February 2020 at the hospital of Hochzirl. Within the first three days after admission, every patient older than 64 years was assigned to the geriatric assessment by the doctor in charge. The assessment was waived if a patient had the following contraindications: (i) lack of consent, (ii) complete independence and no need for assistance in daily living, (iii) stable need for assistance with no prospect of rehabilitation, (iv) terminal illness, or (v) severe dementia and (vi) the current report was not older than 12 weeks. The information on (ii), (iii), (iv) and (v) was obtained from the medical report, produced for each patient on admission to hospital.

The geriatric assessment took approximately 30 to 60 minutes and was performed by a trained graduated nurse following a prespecified procedure (more details are provided below). A test could be skipped if the patient was not able to perform it due to physical or a mental dysfunction. In addition, further information was gathered from the nurse team on the ward in the form of a patient-related questionnaire to collect all the necessary information for ADL. Finally, a document with the results of the geriatric assessment (including ADL) was handed over to the attending physician. The results of the geriatric assessment were gathered by the doctors’ team, and data were collected manually in Microsoft Excel.

#### Outcome variable

Our main variable of interest was ADL, which measures in a standardized way physical independence and, hence, a patient’s need for assistance in everyday life [10]. In particular, we used the Barthel index [11], which consists of ten different everyday tasks: presence/absence of faecal incontinence, presence/absence of urinary incontinence and help needed with grooming, toileting, feeding, transferring (e.g. from chair to bed), walking, dressing, climbing stairs and bathing. Each performance item is rated on a scale with 0, 5, 10 or 15 points, leading to a total score between 0 and 100, with a lower score indicating greater disability [10]. Previous research distinguished between patients who require help in everyday life and those who do not [7, 8, 12, 13]. We followed this approach and created an indicator variable ADL*, which equalled 1 if a patient scored less than or equal to 80 points and zero otherwise [14–16].

#### Explanatory variables

To determine the driving impairments behind ADL, we relied on all test procedures of the geriatric assessment. These included the general health status (i.e., the clinical admission) of the patients as well as information on their mobility, cognitive abilities, nutritional status, urinary incontinence, sensory functions and general psychological situation. In total, our sample included 23 geriatric variables which we divided into *seven categories* (number of variables in parentheses):^1^

- *Mobility (3):* Falling is often caused by mobility problems [3]. To identify the corresponding impairments, we used the Tinetti mobility test (TMT) [17, 18], the timed up and go test (TUG) [19], and a patient’s grip strength (GS) [20]. TMT consists of a balance and a gait test in which 28 points can be achieved [18]. Different manoeuvres and assessment criteria allow early detection of mobility disorders and their underlying limitations. The tasks correspond to movements of daily living, and the treatment of impairments is intended to increase mobility and thus reduce the risk of falls [17]. TUG is a quick test to assess basic functional mobility. At the sign of a supervisor, using everyday walking aids and wearing shoes, a participant must stand up from an armchair, walk three metres, turn around, walk back to the armchair and sit down again. The supervisor times this process [19]. GS is intended to give an indication of body strength and was measured in kPa using a dynamometer from KERN & SOHN (Balingen-Frommern, Germany). The procedure was performed according to a validated protocol [21, 22] in seated position. Upper arm was folded, elbow bent at 90^∘^ and wrist in neutral position. Three measurements were taken with the dominant hand and the mean value was reported.
- *Cognitive function (3):* Recognition and information processing issues may indicate cognitive deficits. In geriatric assessments, cognitive performance was screened with the Mini Mental State Examination (MMSE) [23], the clock completion test (CC) [24], and the money counting test (MC) [25]. The MMSE is a test consisting of eleven questions that distinguishes people based on their likelihood of having a cognitive impairment. The procedure only takes between five and ten minutes and is therefore also suitable for older people suffering from (weak) dementia. In the first part of the test, orientation, memory, and attention are assessed verbally. In the second part, the ability to name and execute verbal and written commands is measured [23].
- *Nutritional status (3):* Older adults often suffer from vitamin and mineral deficiencies [3]. Therefore, as part of the geriatric assessments, nutritional status was evaluated by the Mini Nutritional Assessment (MNA) [26], body mass index (BMI) [27] and fat mass (FM) [28]. Guigoz et al. developed the MNA, a 15-minute questionnaire. It records anthropometric parameters such as BMI or upper arm circumference, general condition in terms of housing situation or skin problems, nutritional habits such as the number of daily meals or food choices, and a self-assessment of malnutrition and health status [26].
- *Incontinence Screening (4):* Urinary incontinence has a negative impact not only on physical health due to an increased risk of infections, falls and death but also on mental health as self-esteem suffers and affected people isolate themselves more often [3, 29]. Furthermore, it is often not mentioned by patients, so it is necessary to observe incontinence directly. In the geriatric assessments, urinary incontinence was recorded by the urinary incontinence score (ICS), the urinary incontinence diagnosis (ICD) and information on a permanent catheter (PC) [21]. Furthermore, we included a urinary incontinence indicator (ICI) variable to indicate whether information on ICS was available. The ICS is a five-question survey that asks about situations in which urine is lost in an uncontrolled way. One point is awarded for each question answered with “yes”. This questionnaire is also used to specify the type of urinary incontinence. Depending on which question is answered positively, the ICD registers urge (captured by the dummy variable ICD_1_), stress (ICD_2_) or mixed urinary incontinence (ICD_3_). ICD_4_ indicates uncertainty about the presence of urinary incontinence, and ICD_0_ suggests no urinary incontinence [21].
- *Sensory function (2):* Carabellese et al. showed that sensory impairments in older people have a negative impact on their social relationships and self-sufficiency [30]. To capture sensory functions, our geriatric assessment incorporated an indicator variable on hypacousia (HC) (hearing abilities) and a categorical variable capturing pain (PAIN) [31].
- *Psychological situation (1):* The most common psychological illness in older people is depression, which is screened for with the Geriatric Depression Scale (GDS) [32]. The GDS consists of 15 simple questions. Due to the short form, little concentration and administration time is needed. For example, the questionnaire asks whether many activities and interests have been dropped or whether it is wonderful to be alive now.
- *Clinical admission status (7):* To account for a patient’s general health status, we included the patient’s age (AGE) and sex (SEX), whether and which type of diabetes mellitus (DM) was diagnosed, and whether medication for diabetes mellitus therapy (DMT), arterial hypertension (AH) or cerebrovascular diseases (CVD) were prescribed prior to the assessment or polypharmacy (PP) occurred.

Table A.1 of Additional file 1 provides a summary of all variables included in the study along with a description of the units of measurement.

#### Descriptive statistics

Our sample included information on the assessments of 326 geriatric patients. This information included ADL along with 23 covariates collected in these assessments (see Table A.1 of Additional file 1). Table 1 reports the corresponding descriptive statistics. Our sample consisted of 74% females. The age of the patients was 80.6±7.4 years and varied between 64 and 99 years. The ADL score for all patients in the sample was 70.2±20.3 points, with a minimum of 10 and a maximum of 100. Furthermore, and according to the 80%-threshold of ADL defined above (i.e., ADL*), we observed that around 69% of all patients needed assistance in everyday life. Regarding other geriatric covariates, we observed body strength (GS) of 38.9±15.8 kPa, a fall risk score (TMT) of 16.7±2.3 and a mobility impairment test (TUG) of 35.8±9.8 seconds. The BMI was 26.7±6.3.

**Table 1:** Descriptive statistics (*N*=326)

Variable		Missing		Statistics		Range	
Abbr.	Name	*N*	Ind.	Mean	s.d.	Min.	Max.
ADL	Activities of daily living	0		70.17	20.27	10	100
ADL ^∗^	ADL indicator variable	0		0.69	0.46	0	1
GS	Grip strength	7	$\checkmark$	38.87	15.75	0	85
TMT	Tinetti mobility test	36	$\checkmark$	16.71	2.27	1	27
TUG	Timed up and go test	38	$\checkmark$	35.76	9.80	7	110
CC	Clock completion test	9	$\checkmark$	2.73	2.93	0	7
MC	Money counting test	75	$\checkmark$	55.09	30.57	18	183
MMSE	Mini Mental State Examination	10	$\checkmark$	25.38	3.93	5	30
BMI	Body mass index	9	$\checkmark$	26.67	6.26	14	59
FM	Fat mass	83	$\checkmark$	36.78	7.42	4	50
MNA	Mini Nutritional Assessment	10	$\checkmark$	19.51	2.98	9	25
ICD_1_	Urge urinary incontinence	9	$\checkmark$	0.38	0.49	0	1
ICD_2_	Stress urinary incontinence			0.04	0.21	0	1
ICD_3_	Mixed urinary incontinence			0.17	0.38	0	1
ICD_4_	Uncertainty about urinary incontinence			0.17	0.38	0	1
ICS	Urinary incontinence score	8	$\checkmark$	1.70	1.35	0	5
PC	Permanent catheter	9	$\checkmark$	0.14	0.34	0	1
ICI	Urinary incontinence indicator	8	$\checkmark$	0.13	0.33	0	1
HC	Hypacousia	11	$\checkmark$	0.50	0.50	0	1
PAIN	Pain	7	$\checkmark$	5.21	1.93	0	10
GDS	Geriatric Depression Scale	11	$\checkmark$	6.13	2.04	0	12
AGE	Age	8	$\checkmark$	80.64	7.37	64	99
AH	Arterial hypertension	0		0.69	0.46	0	1
CVD	Cerebrovascular diseases	3	$\checkmark$	0.16	0.37	0	1
DM_1_	Type 1 diabetes mellitus	0		0.01	0.10	0	1
DM_2_	Type 2 diabetes mellitus			0.22	0.41	0	1
DMTH_1_	Insulin therapy against diabetes mellitus	0		0.08	0.28	0	1
DMTH_2_	Oral therapy against diabetes mellitus			0.07	0.26	0	1
PP	Polypharmacy	3	$\checkmark$	0.82	0.38	0	1
SEX	Sex	0		0.74	0.44	0	1

*Abbreviations*: *N* number of observations; *s.d.* standard deviation; *Ind.* indicator variable

*Notes*: ADL ^∗^ indicates an ADL disability. The subscript informs about the type of impairment. For example, ICD revers to urinary incontinence diagnosis and the subscript indicates the type or urinary incontinence. A detailed variable description is reported in Table A.1 of Additional file 1

Generally, our sample characteristics were comparable to those of previous clinical studies [33, 34]. Bahat et al., for instance, is the study most closely related to the clinical setting. These authors used a sample of 406 patients between 65 and 99 years with an average age of 76.6±7.7 years, a female share of 69.7% and a BMI of 29.7±5.4 [34].

Column 2 of Table 1 provides information about the number of missing values per variable. It shows that missing entries were unevenly distributed across our variables. While we had no missings for ADL, AH, DM, DMT and SEX, we observed a much larger share of missings for TMT (36), TUG (38), MC (75), and FM (83). As described above, missing entries were not completely at random. For instance, it was clear from the outset that some patients were not able to take the TUG or the MC. To account for this nonrandomness, we added an indicator variable for each variable with at least one missing value, with an entry of 1 if the original variable was not collected and zero otherwise. In the table, these variables are indicated by $\checkmark$; subsequently, they are denoted by 1_x_, where “x” indicates a particular variable in the dataset. Furthermore, we applied a standard imputation approach and replaced the missing entries with the mean of the associated variable. In this way, our approach ensured easy interpretation of our parameter estimates and clear identification of missings in our dataset.

### Statistical analysis

For variable selection and model evaluation we proceeded in a two-step approach. In the variable selection process, the least absolute shrinkage and selection operator (LASSO) was applied to identify the most influential geriatric covariates on the numeric ADL. In the second step, as we were solely interested in whether a person was independent from everyday assistance, we transformed ADL to a binary variable and performed logistic regression techniques and receiver operating characteristic (ROC) analysis to evaluate the variable selection of the first step. More specifically, we calculated the area under the ROC curve (AUC) as a measure of discriminatory power. In addition, to easily identify patients with a potential ADL disability, we derived optimal cut-off values for the selected geriatric variables. To test their strength, we used odds-ratios. Finally, an out-of-sample exercise was performed to validate the robustness of our results.

#### Variable selection

To identify the most relevant geriatric covariates driving ADL, we applied LASSO [35]. This method is applicable for high-dimensional data reduction and feature selection as it performs both variable selection and regularization to enhance the detection accuracy and interpretability of the resulting model. The basic intuition behind LASSO is that it forces the sum of the absolute value of the regression coefficients to be less than a fixed value (known as the regularization parameter, henceforth *λ*). As a result, less important coefficients are shrinked to zero, i.e., are excluded from impacting the model. Since optimal selection of the regularization parameter is critical, we used 100-fold cross-validation to detect optimal values of *λ* based on the resulting regression errors. We chose two different *λ*-specifications in the variable selection process [36]. The first one leads to the minimum mean cross-validation error (*λ*_*min*_), and the second one is the largest value of *λ* such that the error is within one standard error of the cross-validated errors for *λ*_*min*_. We refer to this as *λ*_1*se*_.

#### Model evaluation

Based on our definition of neediness in everyday life, we examined the extent to which our geriatric covariates were able to explain our indicator variable ADL* [7, 8, 12, 13]. For this purpose, we used a logistic regression framework and ROC analysis for both *λ*-specifications.

Furthermore, one might be interested in the univariate effects of the selected variables on ADL* and in the cut-off values of these geriatric covariates as they are essential for clinical decision-making. In medical practice, for example, cut-off values support the diagnosis of an impairment and thus the initiation of appropriate therapies or assistance [37]. Of the standard approaches to estimate those cut-off values [37, 38], we used the Youden index, which defines the optimal cut-off as the point maximizing the Youden function, which is the difference between the true positive rate and false positive rate over all possible cut-off values [39–41]. To assess the strength of each optimal cut-off value, we reclassified the geriatric covariate into a binary variable with a value of 1 if an ADL disability was indicated and 0 otherwise. Then, we used the binary covariate as an explanatory variable for ADL* in a logistic regression to determine its odds ratio, i.e., the factor by which the probability of an ADL disability is greater when the binary covariate under consideration indicates such a disability than when it does not.

#### Out-of-sample robustness

In order to validate the robustness of our results, we performed a 100-fold out-of-sample validation exercise. For this purpose, we assigned to each observation a probability of 0.2 of belonging to the out-of-sample subsample and, therefore, a probability of 0.8 to be part of the in-sample data. We performed variable selection (LASSO) and logistic regression based on the observations on the in-sample-group and obtained predictions accuracy’s (AUC values) on ADL* disability for the out-of-sample subsample. The same approach was applied for the univariate analysis. We repeated this procedure 100 times. In doing so, we got a distribution on both the inclusion of the single variables from LASSO procedure and the cut-off values, which served as robustness measures for variable selection and their optimal cut-off value for estimating an ADL* disability. Furthermore, resulted out-of-sample distributions for AUC were used to assess the reliability and robustness of our results.

#### Software and computational details

The statistical analysis was conducted with R version 4.1.1 [42]. For the LASSO variable selection, we used the package glmnet [43], for the ROC analysis we used the package ROCit [44], and for the logistic regressions we used the package car [45].

## Results

In this section, we first describe which variables were selected to explain ADL. Then, we derive the underlying cut-off values and present the evaluation of the selected variables based on their detection accuracy. Finally, we check the out-of-sample robustness of our results.

### Variable selection

We applied LASSO to detect the geriatric covariates that serve as appropriate explanatory variables to describe ADL and cross-validation to estimate the penalty term *λ*.

Using *λ*_1*se*_, nine variables, TUG, TMT, MMSE, MNA, ICD_4_, ICS, and the dummy variables for missing values on TUG (1_*TUG*_), TMT (1_*TMT*_), and MC (1_*MC*_), were selected to explain ADL. In the case of *λ*_*min*_, five additional geriatric covariates were entered into the model: ICD_3_, PC, GDS, AGE and 1_*GS*_. Hence, we included 14 variables to explain ADL in this specification (Table 2).

**Table 2:** Joint detection accuracy of the data-driven selected explanatory variables

Specification	AUC	*K*	Variables								
*λ*_1*se*_	0.89	9	TUG	TMT	1_*TUG*_	1_*TMT*_	MMSE	1_*MC*_	MNA	ICD_4_	ICS
*λ*_*min*_	0.91	14	9 variables from		*λ*_1*se*_ +	1_*GS*_	ICD_3_	PC	GDS	AGE	

*Abbreviations:TUG* timed up and go test; *TMT* Tinetti mobility test; *1*_*TUG*_ missing TUG; *1*_*TMT*_ missing TMT; *MMSE* Mini Mental State Examination; *1*_*MC*_ missing money counting test; *MNA* Mini Nutritional Assessment; *ICD*_4_ urinary incontinence unclear; *ICS* urinary incontinence score; *1*_*GS*_ missing grip strength; *ICD*_3_ mixed urinary incontinence; *PC* permanent catheter; *GDS* Geriatric Depression Scale; *AGE* age

*Notes*: ADL* indicates an ADL disability. AUC denotes the area under the ROC curve and measures the detection accuracy towards ADL*. *K* denotes the number of selected variables

As described above, TUG, TMT and GS are mobility measures. MMSE and MC provide information on cognitive function. Nutritional status is measured by the MNA. ICS and ICD are used to identify the type of urinary incontinence, while PC indicates a permanent catheter. The psychological situation is captured by the GDS and AGE relates to the general health status.

### Model evaluation and cut-off values

To assess the effectiveness of the data-driven selected variables in identifying the need for assistance in everyday life, we applied logistic regression techniques and ROC analysis. We were interested in the detection accuracy as well as the optimal cut-off values of the explanatory variables and their strength to indicate ADL disability.

Table 2 reports the detection accuracy of the jointly used explanatory variables per *λ*-specification. If the nine variables, selected under *λ*_1*se*_, were used jointly to explain ADL*, the need for assistance was recognized with good accuracy (AUC = 0.89). Using the 14 explanatory variables, selected under *λ*_*min*_, functional limitations in everyday life were identified with high accuracy (AUC = 0.91). Therefore, the potential of the joint use of the data-driven selected variables to capture ADL disability was supported by these higher detection accuracies compared to previous literature [7–9, 34, 46] when no prior observed ADL was taken into account [4].

We were also interested in the univariate performance of the variables selected under *λ*_1*se*_, since in the case of Guralnik et al. or Prasitsiriphon and Weber the joint use of variables was only partially able to outperform individual explanatory variables [7, 8]. Furthermore, we calculated cut-off values for each of the selected variables, which served as thresholds to indicate ADL impairment. These thresholds might be very important for clinical purposes [37]. We compared these values with those from previous studies, which were either derived only for mobility measures [8, 12, 13] or are mentioned in the literature without reference to ADL.

Table 3 reports the AUC for each selected variable along with the corresponding cut-off value (CV), the classification commonly used in the literature and the strength of the cut-off value, measured as the odds ratio (OR) with its 95% confidence interval (95%-CI). For example, TUG is able to identify an ADL disability with good accuracy (AUC = 0.85). If patients take 36 seconds or more to complete the TUG, they are significantly 13.4 times more likely to need assistance in daily living than patients who complete the test in less than 36 seconds. This finding is in line with the literature, which assumes mobility impairment from 30 seconds onwards [19].

**Table 3:** Univariate evaluation of the data-driven selected explanatory variables

Variable	AUC	CV	Interpretation	OR	95%-CI
TUG	0.85	36	≤ 10: mobility normal	13.36	7.35 - 24.3
			11-19: little mobility		
			20-29: limited mobility		
			≥ 30: mobility impairment		
TMT	0.82	17	< 20: increased falling risk	13.96	6.89 - 28.3
1_*TUG*_	0.57	1	=1: missing TUG	9.57	2.26 - 40.6
1_*TMT*_	0.57	1	=1: missing TMT	8.95	2.11 - 38.0
MMSE	0.66	27	≤ 17: severe cognitive deficit	2.75	1.69 - 4.47
			≤ 24: cognitive deficit		
1_*MC*_	0.59	1	=1: missing MC	3.31	1.66 - 6.59
MNA	0.63	21	≥ 24: satisfactory	2.38	1.47 - 3.85
			17-23.5: malnutrition risk		
			< 17: poor nutritional status		
ICD_4_	0.61	1	=1: urinary incontinence unclear	15.12	3.61 - 63.4
ICS	0.59	1.70	≥ 1: incontinence probable	2.57	1.59 - 4.15

*Abbreviations*: *TUG* timed up and go test; *TMT* Tinetti mobility test; *1*_*TUG*_ missing TUG; *1*_*TMT*_ missing TMT; *MMSE* Mini Mental State Examination; *1*_*MC*_ missing money counting test; *MNA* Mini Nutritional Assessment; *ICD*_4_ urinary incontinence unclear; *ICS* urinary incontinence score

*Notes*: ADL* indicates an ADL disability. AUC denotes the area under the ROC curve and measures the detection accuracy towards ADL*. CV denotes the cut-off value at which the explanatory variable has to be split to explain ADL*. OR denotes the odds ratio. 95%-CI indicates the 95% confidence interval of the odds ratio

Overall, on a univariate level, the AUC varied between 0.57 (1_*TUG*_ and 1_*TMT*_) and 0.85 (TUG). As expected, the cut-off values of the dummy variables were 1, so it was assumed that patients who were unable to perform the corresponding tests (1_*TUG*_, 1_*TMT*_, 1_*MC*_) or whose incontinence status was unclear (ICD_4_) needed help in everyday life. The cut-off values we calculated for the metric variables were also supported by the literature as patients who were indicated to have an ADL disability also had an impairment in the respective geriatric covariate. The only exception was the cut-off value of the MMSE, according to which patients with a score below 27 already needed help in everyday life, whereas in the literature a cognitive deficit is only assumed at less than 25 points [47–49].

The odds ratios quantified the strength of these cut-off values, which showed that for a patient with a limitation in one of these explanatory variables, the probability of needing assistance in daily living increased by a statistically significant factor between 2.4 (MNA, 95%-CI 1.5–3.9) and 15.1 (ICD_4_, 95%-CI 3.6–63.4). None of the estimated CI intervals overlapped with the null value (OR=1). This can be used as a proxy of statistical significance of the estimated cut-off values.

### Out-of-sample robustness

We checked the robustness of our results using a 100-fold out-of-sample validation exercise. Regardless of the choice *λ*, we found nine variables to be selected in at least 75 out of 100 LASSO replications. These nine variables exactly coincided with those presented in Table 3. ICD_3_, AGE, GDS, and PC were selected in 61–69% using *λ*_*min*_. The remaining variables appeared in not more than 50% of the out-of-sample exercises. On average, 9.57±1.27 variables were selected using *λ*_1*se*_ and 15.19±3.54 using *λ*_*min*_. The AUC and cut-off values of the univariate analysis were also within the range of cross-validation. The cut-off mean values were largely identical to those of the total sample and the range was always within the corresponding categorisation in the literature.

Using *λ*_1*se*_, we obtained a mean AUC 0.87±0.04 ranging between 0.78 and 0.95. Using *λ*_*min*_, we estimated a mean AUC 0.86±0.05 with a minimum of 0.72 and a maximum of 0.95. This clearly indicates that the AUC values reported in Table 2 are in line with our out-of-sample exercise.

## Discussion

Focusing on single dimensions of ADL, Gobbens and van Assen, Duchowny et al. and Prasitsiriphon and Weber used a test of balance, usual walking speed, GS, physical activity, BMI or fatigue to examine functional limitations [8, 12, 13]. A common characteristic of those works was that the selection of the relevant variables had to be made a priori. In contrast, Jonkman et al. and our approach enabled us to examine the effect of geriatric variables on ADL simultaneously, which in turn allowed us to distinguish between more and less influential dimensions of functional limitations. They found that ten variables associated with mobility (GS, gait speed, five-repeated chair stand time), nutritional situation (BMI), psychological situation (depressive symptoms) and general health status (AGE, CVD, DM, chronic obstructive pulmonary disease, arthritis) was critical for the development of an ADL disability in young older people (65–75 years) who had no impairment at baseline [9]. In our clinical setting, we focused on older patients for whom nine geriatric covariates are critical for ADL impairment. These can be categorized into the four health domains of mobility (TUG, TMT, 1_*TUG*_,1_*TMT*_), cognitive function (MMSE, 1_*MC*_), nutritional status (MNA) and urinary incontinence (ICS, ICD_4_). Wojtusiak et al., despite relying on a different methodological approach and using large-scale rather than clinical data, used a very similar variable choice, i.e., emphasizing the role of cognitive functions, age and urinary incontinence for ADL [4]. Despite broad agreement, these results show that different variables are required for targeted ADL detection depending on the research goal and clinical setting.

When evaluating the selected variables for their ability to detect an ADL disability, the highest accuracy was achieved when our selected variables were used jointly (AUC = 0.89 and 0.91, respectively). Regarding the detection power of single influencing dimensions, only the mobility measures (TUG and TMT) indicated functional limitations with good accuracy. This is in line with Guralnik et al. and Jonkman et al., who also examined the detection accuracy of joint use rather than univariate use of their explanatory variables [7, 9]. However, compared to previous literature that used geriatric covariates univariately or jointly [7–9, 34, 46], we found higher detection accuracy, which clearly supports our data-driven variable selection approach.

The cut-off values we obtained from our analysis were broadly in line with the literature. One notable exception was the MMSE, for which previous papers suggested a cognitive deficit at scores below 25 [47–49], while our study found that scores below 27 indicated a functional impairment. However, Perneczky et al. used a more detailed classification of the MMSE, arguing that a cognitive deficit could be excluded for scores of 30 and was doubtful for scores between 26-29 points [50]. According to this classification, our finding with regard to the MMSE seems plausible.

The robustness of our results was validated using an out-of-sample exercise. We found that the AUC values presented in Table 2 are slightly above the mean AUC values of the out-of-sample exercise, but still within one standard deviation. The univariate in-sample AUC values reported in Table 3 are in the range of the AUC values from the out-of-sample cross-validation and the cut-off values are almost identical. This indicates that the presented regularization approach reduced potential overfitting and resulted in stable estimates.

## Conclusions

We used data from a comprehensive geriatric assessment to explain the activities of daily living (ADL) index, which is used to measure functional impairment and older patients’ needs for assistance in everyday life. Empirically, we proposed a data-driven approach that allowed us to employ all patient-related information typically recorded in a geriatric assessment. In particular, we identified nine variables belonging to four groups of impairments that are most influential for ADL: mobility, urinary incontinence, nutritional status and cognitive function. For each of the underlying variables, we derived cut-off values indicating functional impairment and the need for support in everyday tasks. Jointly, these selected variables were able to indicate ADL disability with high accuracy.

Our findings might be of interest for clinical purposes. First, determining ADL requires a comprehensive observation of patients. In some cases, additional information must be obtained from the referring physicians, and the writing of test protocols is time-consuming and causes high administrative costs. Our study proposes a method of approximating ADL based on limited patient information that is typically easy to obtain. Using this information rather than the ADL score may lead to significant cost savings for a hospital. Second, and perhaps more importantly, ongoing monitoring of the main drivers of ADL allows for early detection of limitations in daily living. This seems particularly interesting for general practitioners and physicians in outpatient care, where information on ADL is usually not collected.

## Supplementary Information

**Additional file 1.** Variables of the geriatric assessment by health category.

## Data Availability

The datasets generated and analysed during the current study are not publicly available due to data protection reasons. The R-codes are available from the corresponding author upon reasonable request.

## Abbreviations

ADL: Activities of daily living
AGE: Age
AH: Arterial hypertension
AUC: Area under the ROC curve
BMI: Body mass index
CC: Clock completion test
CVD: Cerebrovascular diseases
DM: Diabetes mellitus
DMT: Diabetes mellitus therapy
FM: Fat mass
GDS: Geriatric Depression Scale
GS: Grip strength
HC: Hypacousia
ICD: Urinary incontinence diagnosis
ICS: Urinary incontinence score
ICI: Urinary incontinence indicator
LASSO: Least absolute shrinkage and selection operator
MC: Money counting test
MMSE: Mini Mental State Examination
MNA: Mini Nutritional Assessment
PAIN: Pain
PC: Permanent catheter
PP: Polypharmacy
ROC: Receiver operating characteristic
SEX: Sex
TMT: Tinetti mobility test
TUG: Timed up and go test

## Footnotes

1 For the sake of brevity, we henceforth refer to these variables as “explanatory variables” or “geriatric covariates”.
