# Supplementary material for: Detecting disabilities in everyday life: evidence from a geriatric assessment
Source: BMC Geriatr. 2022 Aug 31;22:717. doi: 10.1186/s12877-022-03368-x (PMC9429328; doi:10.1186/s12877-022-03368-x)
Supplement: Supplementary file 1 — Additional file 1. Variables of the geriatric assessment by health category. [file 12877_2022_3368_MOESM1_ESM.pdf]

Additional File 1

on the paper *"Detecting Disabilities in Everyday Life: Evidence from a Geriatric Assessment"*
from Cornelius Dzien, Petra Unterberger, Paul Hofmarcher, Hannes Winner and Monika Lechleitner

Table A.1: Variables of the geriatric assessment by health category

**Measure**	**Variable**	**Unit, Range**	**Interpretation**
**Everyday competence**	ADL	0 – 100 points	≤30: largely dependent on care
(outcome variable)		(interval 5)	35 – 80: in need of assistance
			85 – 95: selectively dependent
			100: completely independent
**Mobility**			
– Total body strength	GS	kPa	male <65: low body strength
			female <38: low body strength
– Fall risk	TMT	0 – 28 points	<20: increased risk of falling
– Mobility impairment	TUG	seconds	≤10: normal mobility
			11 – 19: little mobility
			20 – 29: limited mobility
			30: severe mobility impairment
**Cognitive Function**			
– Cognitive impairment	CC	0 – 7 points	>3: cognitive impairment
– Need for health care	MC	0 – 300 seconds	<45: unassisted
			45 – 70: assistance
			>70: substantial
– Cognitive deficit	MMSE	0 – 30 points	≤17: severe cognitive deficit
			≤24: cognitive deficit
**Nutritional Status**			
– Body weight to height ratio	BMI	kg/m^2	<24: malnourished
			24 – 30: normal
			>30: overweight
– Body fat mass	FM	0 – 100 percent	male >25: high FM
			female >36: high FM
– Malnutrition	MNA	0 – 30 points	≥24: satisfactory
			17 – 23.5: malnutrition risk
			<17: poor nutritional status
**Incontinence Screening**			
– Type of urinary incontinence	ICD	0,1,2,3,4	0: no urinary incontinence
			1: urge urinary incontinence
			2: stress urinary incontinence
			3: mixed urinary incontinence
			4: urinary incontinence unclear
– Extent of incontinence	ICS	0 – 5 points	≥1: urinary incontinence
– Permanent catheter	PC	0,1	0: no permanent catheter

Continued on next page

Table A.1: Variables of the geriatric assessment by health category (Continued)

**Measure**	**Variable**	**Unit, Range**	**Interpretation**
			1: permanent catheter
– Availability of ICS due to PC	ICI	0,1	0: ICS available
			1: ICS not available due to PC
**Sensory Function**			
– Hearing ability	HC	0,1	0: normal hearing ability
			1: impaired hearing ability
– Numerical rating scale	PAIN	0 – 10 points	higher value = greater pain
**Psychological Situation**			
– Screening for depression	GDS	0 – 15 points	<6: depression unlikely
			6 – 10: moderate depression likely
			>10: severe depression likely
**Clinical Admission Status**			
– Sex	SEX	0,1	0: male
			1: female
– Age at admission	AGE	years	
– Blood glucose	DM	0,1,2	0: no diagnosed diabetes
(diagnosis DM)			1: type 1 diabetes
			2: type 2 diabetes
– Diabetes mellitus treatment	DMT	0,1,2	0: no treatment
			1: insulin
			2: oral treatment
– Blood pressure	AH	0,1	0: normal blood pressure
			1: increased blood pressure
– Cardiovascular stroke	CVD	0,1	0: no stroke
			1: stroke
– Different medications	PP	0,1	0: <5 different drugs
			1: ≥5 different drugs

Abbreviations: ADL: activities of daily living; GS: grip strength; TMT: Tinetti mobility test; TUG: timed up and go test; CC: clock completion test; MC: money counting test; MMSE: Mini Mental State Examination; BMI: body mass index; FM: fat mass; MNA: Mini Nutritional Assessment; ICD: urinary incontinence diagnosis; ICS: urinary incontinence score; PC: permanent catheter; ICI: urinary incontinence indicator; HC: hypacousia; PAIN: pain; GDS: Geriatric Depression Scale; SEX: sex; AGE: age; DM: diabetes mellitus; DMT: diabetes mellitus therapy; AH: arterial hypertension; CVD: cerebrovascular diseases; PP: polypharmacy.
